# Rotenone Remarkably Attenuates Oxidative Stress, Inflammation, and Fibrosis in Chronic Obstructive Uropathy

**DOI:** 10.1155/2014/670106

**Published:** 2014-07-22

**Authors:** Ying Sun, Yue Zhang, Daqiang Zhao, Guixia Ding, Songming Huang, Aihua Zhang, Zhanjun Jia

**Affiliations:** ^1^Nanjing Key Laboratory of Pediatrics, Nanjing Children's Hospital, Nanjing Medical University, Nanjing 210008, China; ^2^Department of Nephrology, Nanjing Children's Hospital, Nanjing Medical University, Nanjing 210008, China; ^3^Institute of Pediatrics, Nanjing Medical University, Nanjing, China; ^4^Division of Nephrology, Department of Medicine, New York University Langone Medical Center, New York, NY 10016, USA; ^5^Department of Renal Transplantation, The Third Affiliated Hospital, Sun Yat-sen University, 600 Tianhe Road, Tianhe District, Guangzhou 510630, China

## Abstract

Mitochondrial abnormality has been shown in many kidney disease models. However, its role in the pathogenesis of chronic kidney diseases (CKDs) is still uncertain. In present study, a mitochondrial complex I inhibitor rotenone was applied to the mice subjected to unilateral ureteral obstruction (UUO). Following 7-days rotenone treatment, a remarkable attenuation of tubular injury was detected by PAS staining. In line with the improvement of kidney morphology, rotenone remarkably blunted fibrotic response as shown by downregulation of fibronectin (FN), plasminogen activator inhibitor-1 (PAI-1), collagen I, collagen III, and *α*-SMA, paralleled with a substantial decrease of TGF-*β*_1_. Meanwhile, the oxidative stress markers thiobarbituric acid-reactive substances (TBARS) and heme oxygenase 1 (HO-1) and inflammatory markers TNF-*α*, IL-1*β*, and ICAM-1 were markedly decreased. More importantly, the reduction of mitochondrial DNA copy number and mitochondrial NADH dehydrogenase subunit 1 (mtND1) expression in obstructed kidneys was moderately but significantly restored by rotenone, suggesting an amelioration of mitochondrial injury. Collectively, mitochondrial complex I inhibitor rotenone protected kidneys against obstructive injury possibly via inhibition of mitochondrial oxidative stress, inflammation, and fibrosis, suggesting an important role of mitochondrial dysfunction in the pathogenesis of obstructive kidney disease.

## 1. Introduction

Fibrosis is a common event of various forms of chronic kidney diseases (CKDs). The progression of renal fibrosis has been thought as a major pathological process leading to the progressive loss of renal function in CKDs [1–3]. Among those causative factors leading to the renal fibrosis, inflammation and oxidative stress were best characterized [4–7]. In the past decades, although numerous studies have been performed aiming to develop better strategies for treating the CKDs [8, 9], the therapeutic outcome is still unsatisfactory owing to the incomplete understanding of pathological mechanisms.

Interestingly, recent reports indicated an abnormal change of mitochondria in some CKD models including UUO [10–12] and 5/6 nephrectomy [13]. Mitochondria not only are the key source of energy production but also play important roles in mediating the signaling transduction, cell proliferation and the control of cell cycle, cell growth, and cell death [14, 15]. The dysfunction of mitochondria causes ATP depletion, reactive oxygen species (ROS) overproduction, and release of proapoptotic factors like cytochrome C and mitochondrial DNA, which could result in the cell injury via oxidative damage of DNA and protein and apoptotic response and subsequent inflammation and fibrosis [16, 17]. In agreement with these notions, our previous study gave the evidence showing that mitochondrial dysfunction is an early event prior to the occurrence of renal fibrosis in mouse model with aldosterone infusion [18] and the intervention of mitochondrial dysfunction remarkably attenuated renal injury induced by chronic aldosterone infusion. In obstructed kidneys, the mitochondrial abnormality is also proven [10, 12], suggesting an involvement of mitochondrial dysfunction in the pathogenesis of obstructive kidney disease.

UUO is a well-established and widely used model in investigating the mechanisms and therapeutic strategies of renal fibrosis [19]. The mitochondrial abnormality in obstructed kidney might be a cause leading to the renal fibrosis or merely a secondary result of renal injury. To define the role of mitochondrial dysfunction in obstructive kidney injury, we treated the UUO mice with a mitochondrial complex I inhibitor rotenone to determine (1) whether inhibition of mitochondrial complex I can attenuate tubular injury and renal fibrosis in obstructive kidney disease and (2) whether mitochondrial complex I inhibition could affect the oxidative stress and inflammation in this particular model.

## 2. Methods

### 2.1. Animals

C57BL/6J mice were originally purchased from Jackson lab. This mouse colony was propagated at the Nanjing Medical University. In all studies, 3- to 4-month-old male mice were used. All mice were maintained under a 12 : 12 h light-dark cycle (lights on at 6:00 a.m. and lights off at 6:00 p.m.). This study was approved by the Nanjing Medical University Institutional Animal Care and Use Committee.

### 2.2. Establishment of UUO Mouse Model and Rotenone Treatment

Unilateral ureter obstruction was induced as described previously [20]. Briefly, the left ureter was exposed and subsequently ligated with 6.0 silk through a small abdominal incision under the anesthesia with 2.0% isoflurane. The abdomen was closed in two layers. All mice received analgesia (subcutaneous injection of 50 *μ*g/kg buprenorphine (Temgesic, Schering-Plough)) after the surgery. Following the surgery, the jelly diet with or without rotenone at a dose of 500 ppm was given to the UUO mice. The sham control mice were treated with jelly diet without rotenone. After seven-day treatment of rotenone, mice (*N* = 5 per group) were sacrificed and the kidney tissues were harvested for the evaluation of gene and protein expressions and histological analysis.

### 2.3. Tubular Injury Score

Kidney tissues were fixed by direct immersion in 10% formalin for 16 h. Following embedding in paraffin, 4 *μ*m sections were prepared and stained with periodic acid Schiff (PAS) and analyzed with light microscope. The percentage of tubular injury parameters of epithelial flattening, tubular dilatation, and brush border loss was estimated by a pathologist who was blind to the identity of the specimen using a 4-point scale in ten randomly chosen, nonoverlapping fields (200x magnification). Degree of injury was graded onto a scale from 0 to 4: 0 = normal; 1 = mild, involvement of less than 25% of the cortex; 2 = moderate, involvement of 25 to 50% of the cortex; 3 = severe, involvement of 50 to 75% of the cortex; and 4 = extensive damage involving >75% of the cortex.

### 2.4. Immunohistochemistry

Kidneys were fixed with 10% formalin and embedded in paraffin. Kidney sections (4 *μ*m thickness) were incubated in 3% H_2_O_2_ for 15 minutes at room temperature to block endogenous peroxidase activity. After boiling in antigen retrieval solution (1 mmol/L tris-HCl, 0.1 mmol/L EDTA, pH = 8.0) for 15 minutes at high power in a microwave oven, the sections were incubated overnight at 4°C with rabbit anti-collagen I antibody (Cat number: sc-8784, Santa Cruz). After washing with PBS, the secondary antibody was applied and the signal was visualized using an ABC kit (Santa Cruz Biotechnology).

### 2.5. Immunoblotting

The whole kidney was lysed and protein concentration was determined by Coomassie reagent. Proteins (60 *μ*g) from whole kidney lysates were denatured in boiling water for 10 min, separated by SDS-polyacrylamide gel electrophoresis, and transferred onto nitrocellulose membranes. The blots were blocked overnight with 5% nonfat dry milk in tris-buffered saline (TBS), followed by incubation for 1 h with rabbit anti-collagen I (Cat number: sc-8784, Santa Cruz), anti-fibronectin (Cat number: sc-9068, Santa Cruz), anti-*α*-smooth muscle actin (Cat number: sc-32251, Santa Cruz), or anti-HO-1 (Cat number: ab13248, Abcam) at a dilution of 1 : 1000. After being washed with TBS, blots were incubated with a goat anti-horseradish peroxidase-conjugated secondary antibody (1 : 1000 dilution) and visualized with ECL kits (Amersham, Piscataway, NJ, USA).

### 2.6. qRT-PCR

Total RNA isolation and reverse transcription were performed as previously described [21]. Total DNA from kidney was isolated using the DNeasy Tissue Kit (Invitrogen, Carlsbad, CA). The mRNA and mtDNA copy numbers were detected by qRT-PCR. Oligonucleotides were designed using Primer3 software (available at http://frodo.wi.mit.edu/primer3/) and the sequences are shown in Table 1. qRT-PCR amplification was performed using the SYBR Green Master Mix (Applied Biosystems, Warrington, UK) and the PRISM 7500 Real-Time PCR Detection System (Applied Biosystems, Foster City, CA, USA). Cycling conditions were 95°C for 10 min, followed by 40 repeats of 95°C for 15 s and 60°C for 1 min.

### 2.7. Measurement of Thiobarbituric Acid-Reactive Substances

The measurement of plasma thiobarbituric acid-reactive substances (TBARS) was based on the formation of malondialdehyde by using a commercially available TBARS Assay kit (Cat number: 10009055; Cayman Chemical) according to the manufacturer's instructions.

### 2.8. Enzyme Immunoassay

The kidney tissue was homogenized in phosphate-buffered saline and then centrifuged for 5 min at 10,000 r.p.m. The supernatant was diluted 1 : 50 with enzyme immunoassay buffer. Concentrations of TGF-*β*_1_ were determined by enzyme immunoassay according to manufacturer's instructions (Cat number: ab119557, Abcam). The kidney content of TNF-*α* and IL-1*β* was measured using the ELISA kits (TNF-*α*: Cat number: 559732, BD OptEIA, BD Bioscience; IL-1*β*: Cat number: ab100704, Abcam).

### 2.9. Statistical Analysis

All values are presented as mean ± SE. Statistical analysis was performed using Student's *t*-test or two-way ANOVA. Differences were considered to be significant when *P* < 0.05.

## 3. Results

### 3.1. Effect of Rotenone Treatment on Renal Structural Change in Obstructed Kidneys

**Figure 1:**
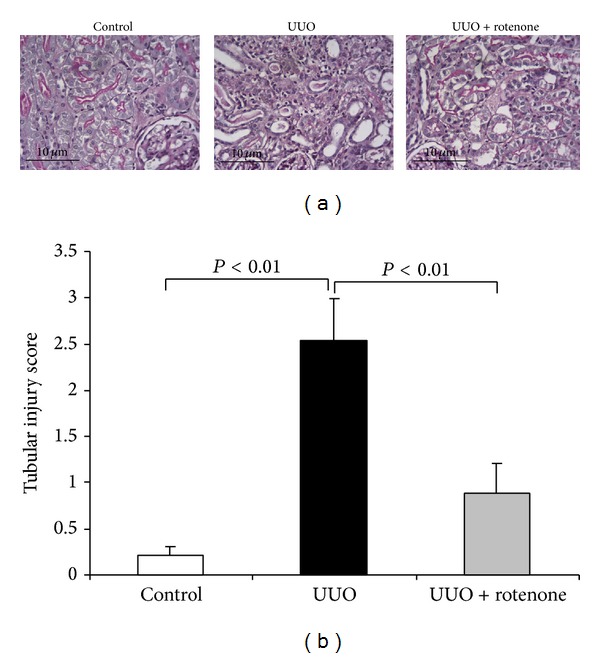
Rotenone treatment attenuated renal tubular injury. (a) Representative images of renal PAS staining in control and UUO mice with or without rotenone treatment. (b) Score of tubular injury. *N* = 5 in each group. Data are means ± SE.

Following 7-day ureteral obstruction, the PAS staining indicated marked tubular structure damage as shown by the loss of brush border, epithelial cell atrophy and flattening, and tubular lumen dilation (Figure 1(a)). Strikingly, 7-day rotenone treatment remarkably attenuated all these morphological abnormalities (Figure 1(a)). Tubular injury score analysis also demonstrated a robust improvement of tubular injury as shown by Figure 1(b).

### 3.2. Effect of Rotenone Treatment on the Oxidative Stress Level in Obstructed Kidneys

**Figure 2:**
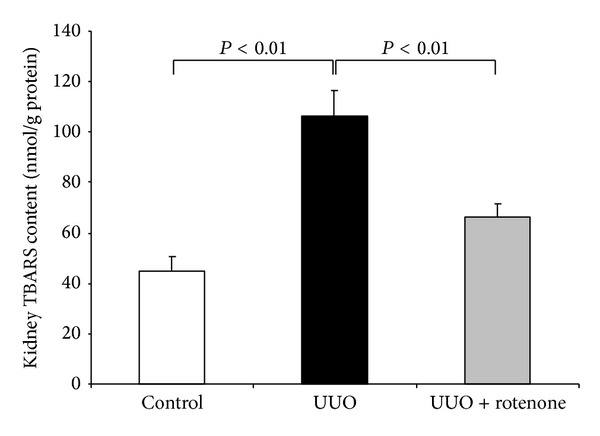
Rotenone treatment blocked the increase of TBARS in obstructed kidneys. Oxidative stress marker TBARS in kidneys was measured using a commercial kit. *N* = 5 in each group. Data are means ± SE.

**Figure 3:**
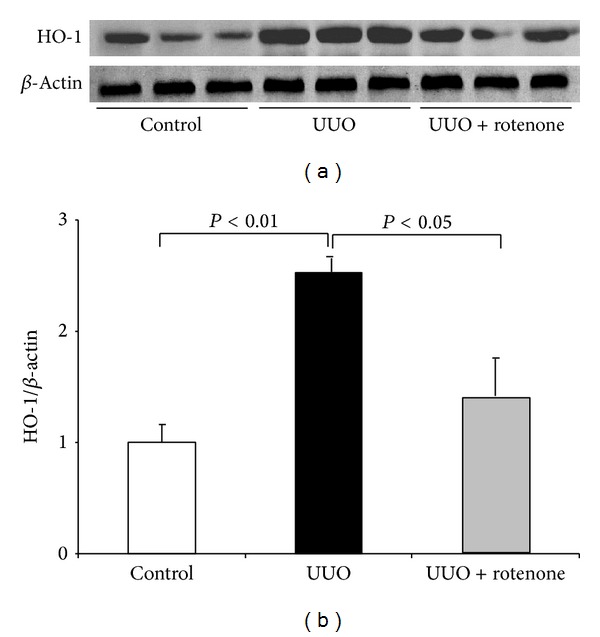
Rotenone treatment blunted HO-1 induction in obstructed kidneys. (a) Oxidative stress marker HO-1 was determined by Western blotting, and *β*-actin was used as loading control. (b) Densitometry of Western blots. *N* = 5 in each group. Data are means ± SE.

Dysfunctional mitochondria serve as important source of ROS production. By blockade of mitochondrial complex I, upregulation of oxidative stress marker HO-1 was significantly blocked in obstructed kidneys (Figures 3(a) and 3(b)). To further validate this antioxidative effect of rotenone in this model, we examined TBARS level using a commercial kit. As expected, the increased TBARS content in obstructed kidneys was strikingly reduced by rotenone administration (Figure 2).

### 3.3. Effect of Rotenone Treatment on Inflammatory Response in Obstructed Kidneys

**Figure 4:**
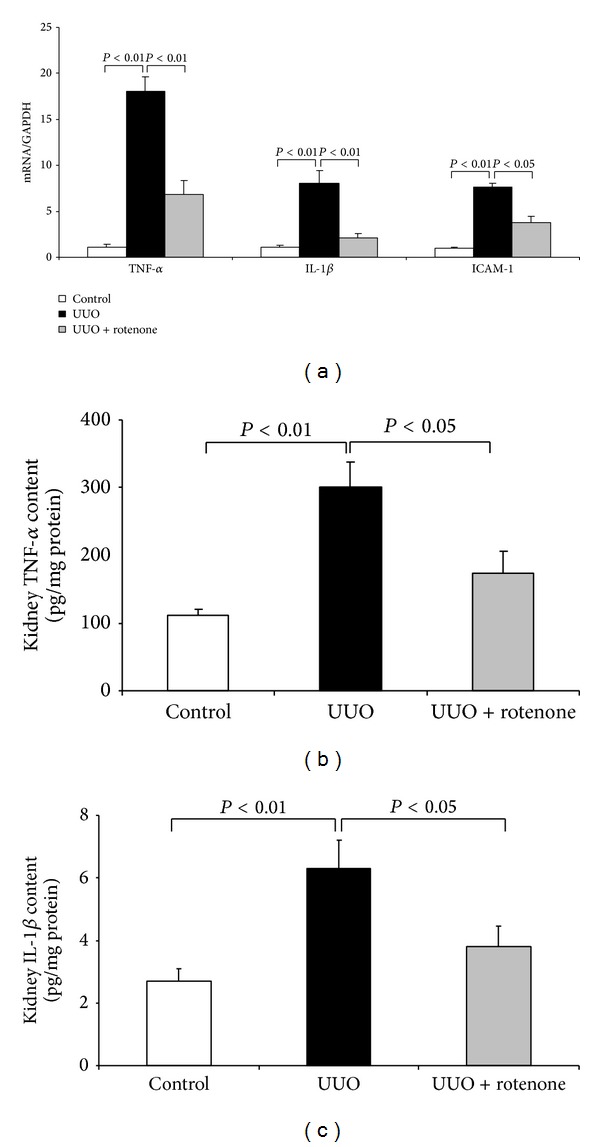
Rotenone treatment blunted inflammatory response in obstructed kidneys. (a) qRT-PCR analysis of TNF-*α*, IL-1*β*, and ICAM-1 mRNA expressions. (b) ELISA analysis of renal TNF-*α* protein level. (c) ELISA analysis of renal IL-1*β* protein level. *N* = 5 in each group. Data are means ± SE.

UUO is also an inflammatory kidney disease model with remarkably augmented inflammation from both infiltrating cells and resident cells. By qRT-PCR, we found that the inflammatory markers TNF-*α*, IL-1*β*, and ICAM-1 were markedly elevated following 7-day ureteral obstruction, and such increments were robustly abolished or attenuated by rotenone administration (Figure 4(a)). By ELISA, we further confirmed the protein regulation of TNF-*α* and IL-1*β* following rotenone treatment (Figures 4(b) and 4(c)).

### 3.4. Effect of Rotenone Treatment on the Fibrotic Markers in Obstructed Kidneys

**Figure 5:**
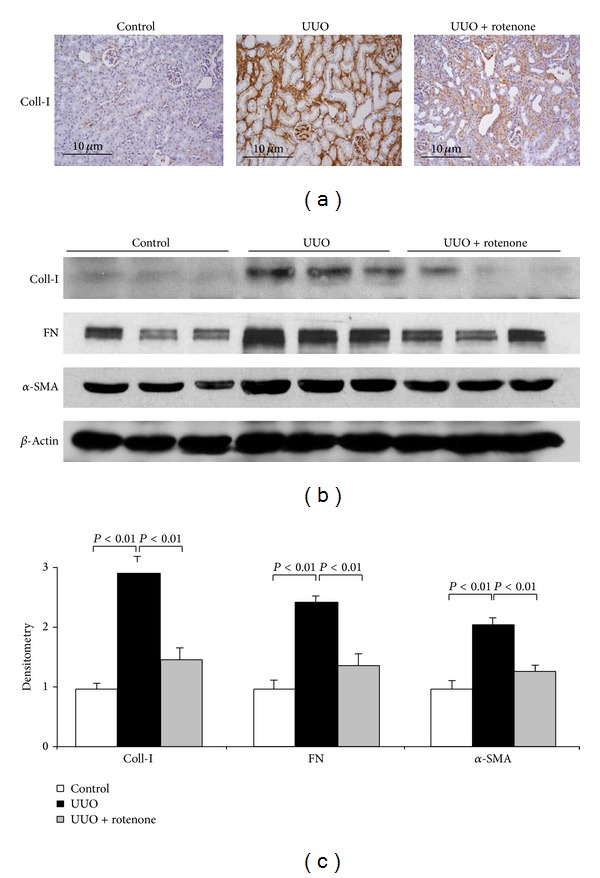
Rotenone treatment reduced protein expression of fibrotic markers in obstructed kidneys. (a) Immunohistochemistry of collagen I. (b) Fibrotic markers of collagen I, FN, and *α*-SMA were determined by Western blotting, and *β*-actin was used as loading control. (c) Densitometry of Western blots. *N* = 5 in each group. Data are means ± SE.

**Figure 6:**
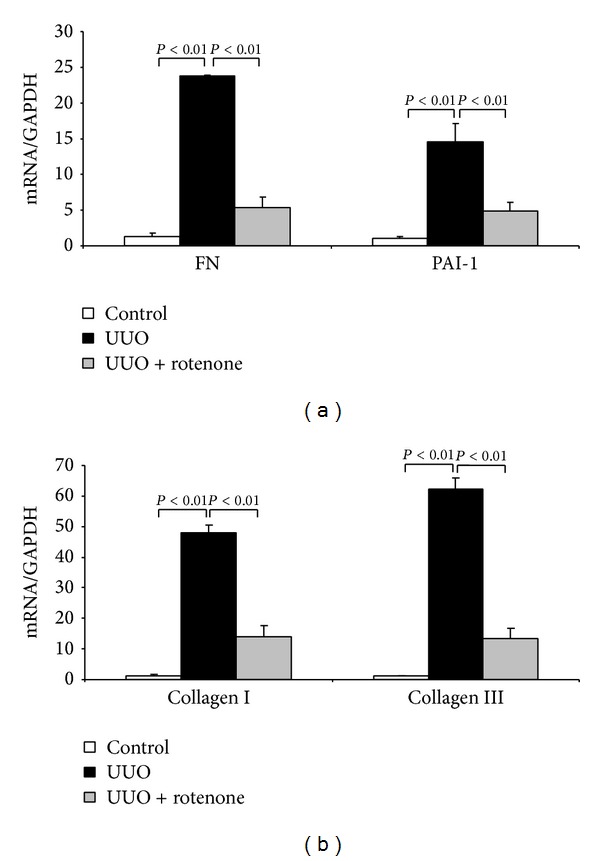
Rotenone treatment reduced mRNA expression of fibrotic markers in obstructed kidneys. (a) qRT-PCR analysis of FN and PAI-1 mRNA levels. (b) qRT-PCR analysis of collagen I and collagen III mRNA levels. *N* = 5 in each group. Data are means ± SE.

Fibrosis is the known pathological phenomenon in UUO animal models. To evaluate the effect of rotenone treatment on the fibrosis in this model, we examined protein and mRNA expressions of cellular matrix components including collagen I, collagen III, FN, PAI-1, and the fibroblast cell marker *α*-SMA. Strikingly, both protein (Figures 5(a)–5(c)) and mRNA (Figures 6(a) and 6(b)) expressions of these markers were robustly downregulated by rotenone treatment. These findings demonstrated an antifibrotic role of rotenone in chronic obstructive kidney disease.

### 3.5. Effect of Rotenone Treatment on TGF-*β*_1_ Expression in Obstructed Kidneys

**Figure 7:**
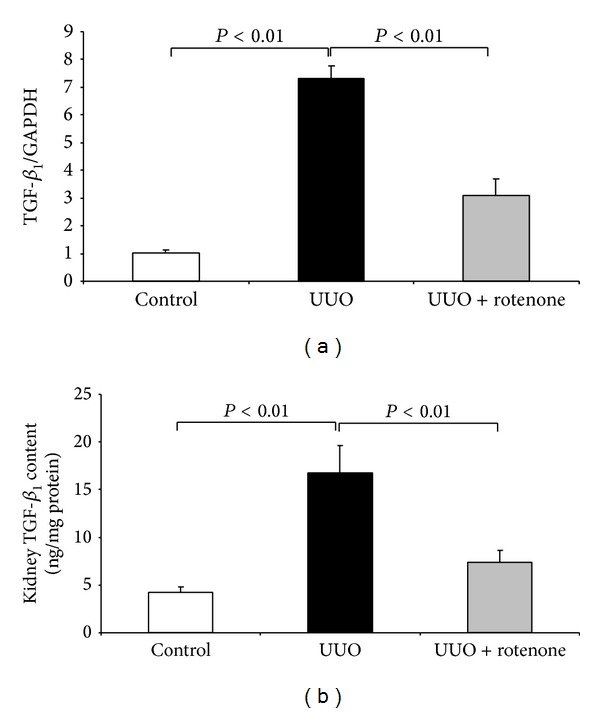
Rotenone treatment decreased renal TGF-*β*_1_ induction in obstructed kidneys. (a) qRT-PCR analysis of renal TGF-*β*_1_ mRNA expression. (b) ELISA analysis of renal TGF-*β*_1_ protein expression. *N* = 5 in each group. Data are means ± SE.

TGF-*β* is a known profibrotic factor in mediating the fibrotic process in obstructive kidney disease. To evaluate rotenone effect on this important fibrotic factor in this UUO model, we examined mRNA and protein levels of TGF-*β*_1_ using qRT-PCR and ELISA, respectively. As shown by the data, both mRNA and protein levels of TGF-*β*_1_ were significantly blunted by rotenone (Figures 7(a) and 7(b)).

### 3.6. Effects of Rotenone Treatment on Mitochondrial DNA Copy Number and mtND1 Expression

**Figure 8:**
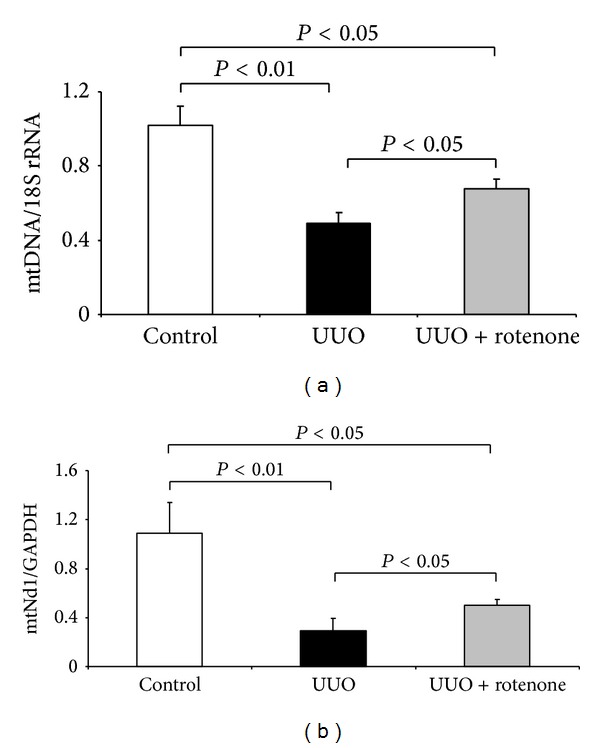
Rotenone treatment partially reversed the reduction of mtDNA copy number and mtND1 expression in obstructed kidneys. (a) mtDNA copy number was determined by qRT-PCR. (b) mtND1 expression was determined by qRT-PCR. *N* = 5 in each group. Data are means ± SE.

To evaluate the mitochondrial abnormality, we measured mtDNA copy number and mtND1 expression. As shown by Figures 8(a) and 8(b), kidney obstruction remarkably reduced both indices, indicating a severe mitochondrial abnormality. After 7-day rotenone administration, reduction of mtDNA copy number and mtND1 expression was moderately but significantly restored (Figures 8(a) and 8(b)), suggesting amelioration of mitochondrial abnormality.

## 4. Discussion

The prevalence of CKD is rapidly rising with the increments of various insults leading to the kidney injury. Among those insults, diabetes and hypertension have become the major ones resulting in the CKD [22, 23]. In the kidneys of CKD patients, the inflammation, oxidative stress, and fibrosis are the common pathological manifestations and form a positive feedback loop to promote progressive renal injury and function loss [4–7]. In the past decades, although the researchers and nephrologists made substantial efforts in understanding the pathogenesis of CKDs, the current management strategies are still ineffective at stopping disease progression. This situation raised an urgent request to better understand the pathogenic mechanisms of CKDs.

Mitochondria are key cellular organelles in determining the cell fate. The dysfunction of mitochondria plays a pathogenic role in chronic heart failure [24, 25] and some central nervous diseases [26]. In kidney, recent reports indicated a potential involvement of mitochondrial dysfunction in mediating the development and progression of CKDs [10–13]. In present study, we applied a mitochondrial complex I inhibitor to the mice subjected to UUO and observed a remarkable amelioration of kidney injury.

Mitochondrial damage not only results in the reduction of ATP level but also leads to the excessive production of ROS, which directly causes cellular injury and subsequently promotes mitochondrial dysfunction [27, 28]. Because the mitochondrial abnormality is a known phenomenon in UUO kidneys [10, 12, 29], in present study, we tested the efficacy of mitochondrial complex I inhibitor rotenone on the oxidative stress in obstructed kidneys and found that rotenone treatment strikingly reduced kidney TBARS content and HO-1 expression. These results demonstrated that inhibiting the activity of dysfunctional mitochondria definitely inhibited ROS overproduction, suggesting a critical role of mitochondrial dysfunction in mediating ROS overproduction in obstructed kidneys. More importantly, with the blockade of mitochondria-originated oxidative stress, the renal tubular injury was also significantly improved as determined by PAS staining, which suggested that mitochondrial oxidative stress served as a pathogenic factor in this pathological process.

Inflammation plays a detrimental role in the occurrence and development of kidney injury in various kidney diseases including CKDs. Both infiltrating inflammatory cells and renal resident cells contribute to inflammatory response in obstructive kidney disease [19, 30–32]. However, the detailed mechanisms leading to the inflammation in this model are still poorly understood. In present study, inhibition of mitochondrial complex I by rotenone led to a marked amelioration of inflammation as shown by significant suppression of proinflammatory cytokines, such as TNF-*α*, IL-1*β*, and ICAM-1. These results suggested that mitochondrial dysfunction may play an important role in mediating the inflammatory response in obstructed kidneys. Also, a number of evidence demonstrated that oxidative stress could trigger the inflammatory response in many pathological processes [33, 34]. In present study, in line with the inhibition of oxidative stress by rotenone, the inflammatory response was also effectively blunted, indicating that mitochondria-derived oxidative stress may be a causative factor in triggering inflammation in obstructive kidney disease.

Fibrosis is a common outcome of all forms of renal injury. Fibrosis, as evidenced by the accumulation of extracellular matrix (ECM) [3] and cellular phenotypic alteration, was thought as a key player in the loss of renal function. Fibrosis not only is a result of various insults, such as inflammation and oxidative stress, but also contributes to the induction of inflammation and oxidative stress, forming a positive feedback loop. In agreement with this notion, blockade of mitochondrial oxidative stress and inflammation by rotenone was paralleled with the attenuation of fibrosis. To further investigate the potential mechanism of this antifibrotic action, we examined TGF-*β*_1_, a key contributor of fibrosis in UUO [35, 36] and many other CDK models [37], and found that TGF-*β*_1_ was decreased by rotenone at both mRNA and protein levels. These data suggested that the antifibrotic action of rotenone is possibly through its blockade of TGF-*β*_1_, at least to some extent. Reduction of TGF-*β*_1_ could be secondary to the attenuation of oxidative stress and inflammation because both ROS [38] and inflammatory cytokines [39] have been shown to be responsible for the TGF-*β*_1_ induction.

In summary, present study demonstrated that inhibiting the activity of dysfunctional mitochondria in obstructed kidney by a mitochondrial complex I inhibitor significantly attenuated kidney injury in parallel with the blockade of oxidative stress, inflammation, and fibrotic response. These results suggested a causative role of mitochondrial dysfunction in mediating the obstructive kidney injury. And a pathogenic loop formed by mitochondrial oxidative stress, inflammation, and fibrosis may exist in this pathological process. Targeting mitochondrial dysfunction may serve as a novel therapeutic strategy for the treatment of obstructive kidney disease and other CKDs.

## Figures and Tables

**Table 1:** Sequences of primers for real-time PCR.

Gene	Primer sequence
GAPDH	5′-gtcttcactaccatggagaagg-3′
	5′-tcatggatgaccttggccag-3′

TNF-*α*	5′-tccccaaagggatgagaag-3′
	5′-cacttggtggtttgctacga-3′

IL-1*β*	5′-actgtgaaatgccaccttttg-3′
	5′-tgttgatgtgctgctgtgag-3′

ICAM-1	5′-cgcttccgctaccatcac-3′
	5′-ggcggctcagtatctcctc-3′

PAI-1	5′-cacgctacttcctcctcaag-3′
	5′-ctctgtcttcatcagctggc-3′

FN	5′-cgtggagcaagaaggacaa-3′
	5′-gtgagtctgcggttggtaaa-3′

Collagen I	5′-ccggctcctgctcctctt-3′
	5′-ttgcacgtcatcgcacac-3′

Collagen III	5′-tggtttcttctcacccttctt-3′
	5′-caaatgggatctctgggttg-3′

TGF-*β*_1_	5′-tacgcctgagtggctgtctt-3′
	5′-cgtggagtttgttatctttgct-3′

mtDNA	5′-atcctcccaggatttggaat-3′
	5′-accggtaggaattgcgataa-3′

18S rRNA	5′-ttcggaactgaggccatgatt-3′
	5′-tttcgctctggtccgtcttg-3′

mtND1	5′-aatcgccatagccttcctaacat-3′
	5′-ggcgtctgcaaatggttgtaa-3′
